# Gut Microbiota‐Derived Indole‐3‐Acetaldehyde Contributes to the Disease‐Alleviating Effects of Kurarinone in Experimental Autoimmune Encephalomyelitis

**DOI:** 10.1002/advs.78358

**Published:** 2026-10-15

**Authors:** Xinyan Han, Jiaqi Ding, Zonghao Pan, Weitai He, Xue Kang, Cong Zhao, Yi Li, Di Wu, Hongzeng Li, Kaixi Ren, Jiaji Lin, Jun Guo

**Affiliations:** ^1^ Department of Neurology Tangdu Hospital The Fourth Military Medical University Xi'an China; ^2^ Department of Neurology Airforce Medical Center of PLA Beijing China

**Keywords:** akkermansia muciniphila, aryl hydrocarbon receptor, experimental autoimmune encephalomyelitis, indole‐3‐acetaldehyde, kurarinone, multiple sclerosis

## Abstract

Multiple sclerosis (MS) is a central nervous system autoimmune disease primarily driven by Th1 and Th17 cells, whose onset and progression are closely associated with gut microbiota. Kurarinone (KU), an active component from *Radix Sophora Flavescens*, exhibits anti‐inflammatory, immunoregulatory and gut microbiota‐modulating properties. However, whether KU alleviates MS severity via a gut microbiota‐mediated mechanism remains unclear. Here, we demonstrate that KU ameliorates experimental autoimmune encephalomyelitis (EAE)‐an animal model of MS‐by modulating the gut microbiota, particularly via enriching *Akkermansia muciniphila*, and *A. muciniphila* is essential for the protective effects of KU in EAE. Furthermore, KU elevates levels of the tryptophan metabolite indole‐3‐acetaldehyde (IAAld). IAAld levels are positively correlated with the relative abundance of *A. muciniphila* and negatively correlated with EAE severity. In vitro, *A. muciniphila* alone is sufficient to facilitate tryptophan metabolism to produce IAAld. IAAld treatment ameliorates EAE and reduces the frequencies of pathogenic Th1 and Th17 cells in mice. Mechanistically, IAAld activates the aryl hydrocarbon receptor (AhR) and suppresses JAK/STAT signaling, thereby dampening Th1 and Th17 differentiation; these effects are abolished following AhR blockade. Collectively, this study identifies a novel mechanism by which KU mitigates EAE via the gut metabolite IAAld‐AhR‐immune axis and unveils potential therapeutic targets for MS.

## Graphical abstract

Kurarinone ameliorates EAE via the gut microbiota‐indole‐3‐acetaldehyde‐AhR axis. Kurarinone reshapes gut microbiota and increases *Akkermansia muciniphila*, which promotes intestinal indole‐3‐acetaldehyde production. Indole‐3‐acetaldehyde crosses the intestinal barrier, activates AhR in CD4^+^ T cells, upregulates SOCS1/3, inhibits JAK2 and STAT3/4 phosphorylation, restrains Th1/Th17 differentiation, and alleviates CNS inflammation and demyelination.

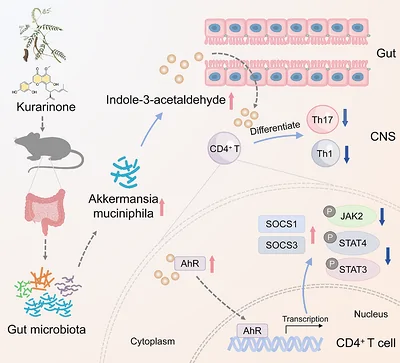

## 1 Introduction

Multiple sclerosis (MS) is a chronic autoimmune disease characterized by demyelination and inflammation in the central nervous system (CNS), and it is the most common cause of non‐traumatic disability in the young and middle‐aged population [1, 2]. To date, the etiology and pathogenesis of MS remain unclear. Existing evidence suggests that the CD4^+^ T cell subsets Th1 and Th17 play vital roles in the disease onset and progression [3, 4]. Cytokines such as interferon‐gamma (IFNγ), secreted by Th1 cells, and interleukin 17A (IL‐17A), secreted by Th17 cells, may promote pro‐inflammatory immune responses, leading to demyelinating lesions in the CNS [5, 6]. Therefore, suppressing the differentiation of naive CD4^+^ T cells into Th1 and Th17 cells has become a critical treatment strategy for MS.

The gut microbiota, a vast and complex microbial community in the human body, is closely linked to the host's health and disease [7, 8]. Increasing evidence has shown that gut microbiota imbalance is implicated in various autoimmune diseases, including MS [9, 10]. In MS patients, the gut microbiota differs from that of healthy individuals, and the abundances of several taxa, such as *Akkermansia muciniphila* and *Collinsella*, are associated with disease severity [11, 12]. Meanwhile, gut microbiota alterations in MS affect the immune responses through multiple pathways, including modulating the differentiation and function of Th1 and Th17 cells [13, 14]. Similar phenomena are observed in animal experiments. Remodeling the gut microbiome with antibiotic treatment or fecal microbiota transplantation (FMT) can regulate Th1 and Th17 cells and exert immunomodulatory effects in experimental autoimmune encephalomyelitis (EAE) mice, the classic animal model of MS [15, 16]. Further studies indicate that these effects may be mediated by gut microbial metabolites, such as short‐chain fatty acids and indole derivatives [11, 16, 17, 18]. Thus, modulating gut microbiota and its metabolites may provide a novel therapeutic strategy for EAE, given their influence on the host's immune system.

Traditional Chinese medicine has been used for disease prevention and treatment for thousands of years with well‐proven efficacy and safety. Mounting modern evidence demonstrates that natural medicines exert therapeutic effects in autoimmune diseases by regulating gut microbiota homeostasis and restoring the host's immune balance [16, 19]. Kurarinone (KU), a major bioactive component of the traditional Chinese medicine *Radix Sophora Flavescens*, exhibits anti‐inflammatory, antioxidant, and immunoregulatory activities that offer new possibilities for treating autoimmune diseases [20]. Previous studies have shown that KU effectively reverses aberrant immune cell profiles in autoimmune uveitis, particularly by reducing Th1 and Th17 cell proportions and restoring the Th17/Treg balance [21]. Meanwhile, KU attenuates inflammatory responses in animal models of inflammatory bowel disease and autoimmune hepatitis by suppressing the cGAS‐STING signaling pathway [20, 22]. In addition, KU treatment inhibits the Th17 cell responses and simultaneously normalizes the gut microbial composition in mice with colitis [23]. Notably, KU effectively ameliorates EAE by suppressing Th1 and Th17 cell differentiation, thus exhibiting promising therapeutic potential for MS [24]. Nevertheless, the precise mechanism by which KU exerts its protective effects against EAE remains largely unclarified. Given the pivotal role of the gut microbiota and its metabolites in modifying Th1‐ and Th17‐dominated autoimmune inflammation, this study primarily aims to explore the potential contribution of gut microbial metabolites to KU's therapeutic effects in EAE.

In this study, we confirmed that oral KU treatment effectively alleviated EAE and simultaneously altered the composition of gut microbiota in EAE mice. Antibiotic depletion and fecal microbiota transplantation (FMT) experiments verified the essential role of gut microbiota in modulating EAE severity. Furthermore, KU enriched *A. muciniphila* and elevated the level of its metabolite indole‐3‐acetaldehyde (IAAld). In vivo experiments showed that oral administration of IAAld recapitulated the therapeutic efficacy of KU in EAE mice. In vitro assays revealed that IAAld activated the aryl hydrocarbon receptor (AhR). Activated AhR suppresses JAK2 phosphorylation, promotes SOCS1 and SOCS3 protein expression, reduces STAT4 and STAT3 phosphorylation and consequently inhibits Th1 and Th17 differentiation. AhR inhibition significantly reversed the protective effect of IAAld in EAE mice. Overall, our research identifies a novel mechanism by which the gut microbiota‐derived indole‐3‐acetaldehyde‐AhR‐immune axis contributes to the disease‐alleviating effects of KU against EAE, providing potential therapeutic targets for MS.

## 2 Results

### 2.1 KU Suppresses Th1 and Th17 Cell Proportions and Mitigates EAE

A previous study has demonstrated that KU at a dosage of 100 mg kg^−1^ effectively ameliorates EAE progression in mice [24]. Accordingly, this dose was adopted in the present study, and KU was orally administered at 100 mg kg^−1^ starting from EAE onset (11 days post‐immunization, 11 dpi; Figure 1A). Compared with the vehicle‐treated EAE mice, KU treatment significantly improved clinical scores (Figure 1B; *p <* 0.05 and *p <* 0.01), decreased disease incidence (Figure 1C; 21 dpi, 75% *vs* 100%), and reduced both cumulative and peak clinical scores (Figure 1D; *p <* 0.05 and *p <* 0.001). Consistently, hematoxylin‐eosin (HE) and luxol fast blue (LFB) staining of spinal cord tissues revealed alleviated inflammatory infiltration and attenuated demyelination in the KU‐treated group (Figure 1E,F; *p <* 0.01 *vs* vehicle‐treated mice). We further analyzed mononuclear cells (MNCs) infiltration and immune cell populations in the CNS and spleen. Flow cytometry results showed that the proportions of CD4^+^, CD4^+^IFNγ^+^ Th1, and CD4^+^IL‐17A^+^ Th17 cells in both the CNS and spleen were significantly decreased after KU treatment (Figure 1G,I,J; *p <* 0.01 and *p <* 0.001), and similar alterations in the numbers of CD3^+^ and CD4^+^ cells in the CNS MNCs were observed (Figure 1H; *p <* 0.001). At the molecular level, KU significantly downregulated the mRNA expression of Th1‐ and Th17‐specific transcription factors *T‐bet* and *RORγt* in spinal cord tissues, accompanied by reduced serum levels of IFNγ and IL‐17A (Figure 1K,L; *p <* 0.05, *p <* 0.01, and *p <* 0.01). In addition, KU treatment decreased the proportions of CD8^+^ T cells, CD19^+^ B cells, and NK1.1^+^ natural killer cells in the CNS and spleen (Figure S2; *p <* 0.05, *p <* 0.01, and *p <* 0.001). Collectively, these findings confirmed KU's therapeutic potential in EAE through inhibiting inflammatory responses primarily driven by Th1 and Th17 cells in the CNS and the periphery.

**FIGURE 1:**
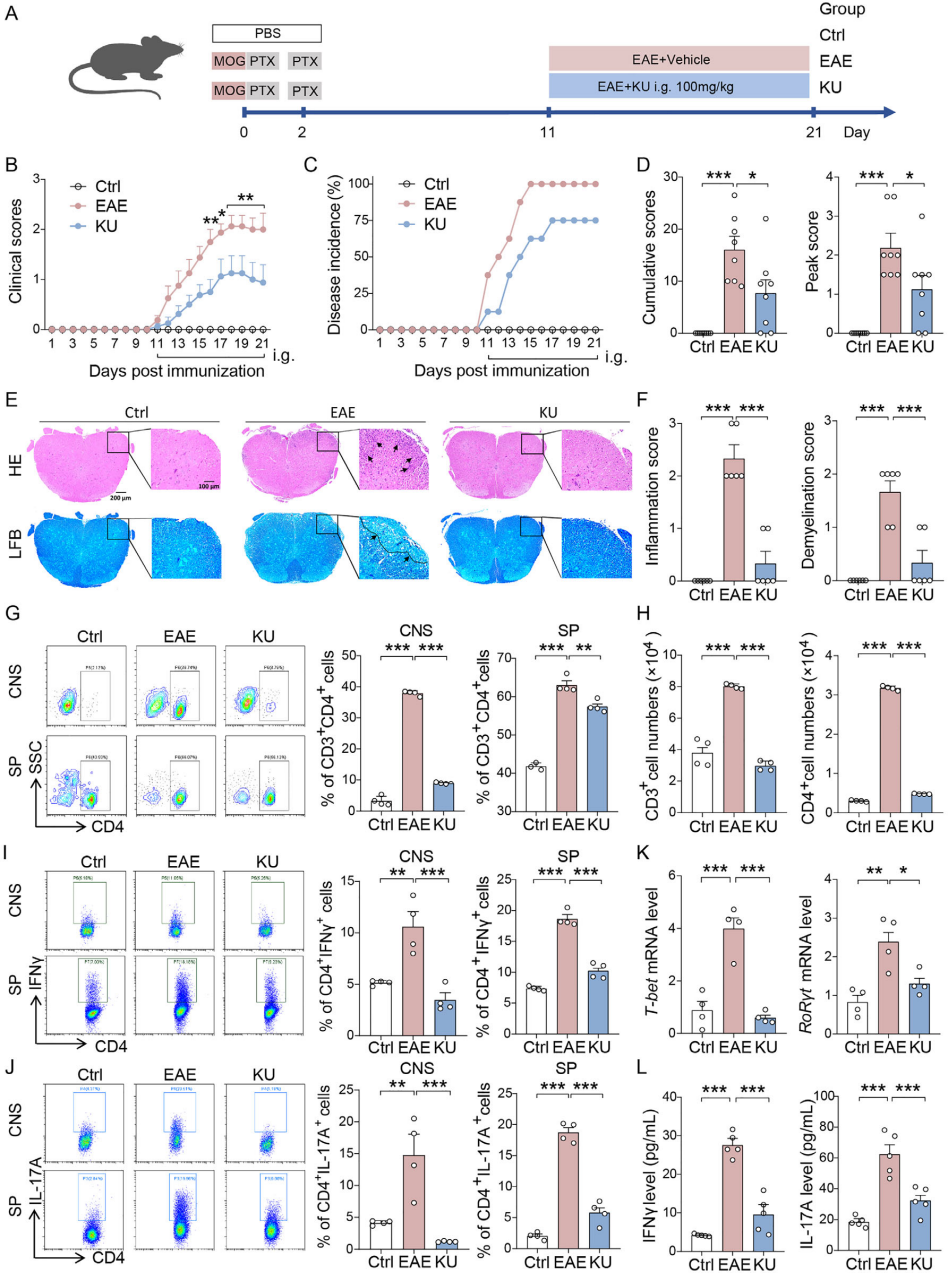
KU treatment mitigates EAE and reduces Th1 and Th17 cell proportions in mice. (A) Schematic timeline of EAE induction and KU administration (100 mg kg^−1^, intragastric). Mice were immunized with MOG_35‐55_ peptide, and KU or vehicle was administered from 11 to 21 dpi. (B) Line graph of clinical scores in the control (Ctrl), vehicle‐treated EAE, and KU‐treated EAE groups (*n =* 8 per group). (C) Disease incidence curves of EAE mice. (D) Cumulative clinical scores and peak score in each group. (E) Representative HE staining and LFB staining of spinal cord from the Ctrl, vehicle‐treated EAE, and KU‐treated EAE groups. (F) Inflammation and demyelination scores based on HE and LFB staining (*n =* 6 per group). (G) The proportion of CD3^+^CD4^+^ T cells in the CNS and spleen (SP) (*n =* 3–4 per group). (H) Absolute counts of CD3^+^ and CD4^+^ T cells in the CNS of each group. (I) The proportion of CD4^+^IFNγ^+^ (Th1) cells in the CNS and SP. (J) The proportion of CD4^+^IL‐17A^+^ (Th17) cells in the CNS and SP. (K) Relative mRNA expression of *T‐bet* and *RORγt* in the spinal cord measured by qPCR. (L) ELISA detection of serum IFNγ and IL‐17A concentrations in each group. Data are presented as mean ± SEM; *p < 0.05, **p < 0.01, ***p < 0.001 vs. vehicle‐treated EAE.

### 2.2 The Therapeutic Effect of KU in EAE is Dependent on the Gut Microbiota

To investigate whether KU alters the gut microbiota composition in EAE mice, we performed metagenomic sequencing on fecal samples collected from the control, vehicle‐treated EAE, and KU‐treated EAE groups. No significant difference in gut microbiota composition was observed between the control group and the vehicle‐treated EAE group, but the α‐diversity of the gut microbiota in the KU‐treated EAE group was significantly lower than that in the vehicle‐treated EAE group, which was attributed to the antibacterial effect of KU [25] (Figure 2A). Principal coordinate analysis (PCoA) showed clear separation of microbial profiles among the Ctrl, EAE, and KU groups, indicating substantial differences in intestinal microbiota composition (Figure 2B). At the phylum level, vehicle‐treated EAE mice exhibited reduced relative abundance of *Bacteroidota* and elevated *Bacillota*. Conversely, KU intervention effectively reversed these EAE‐induced shifts in gut microbiota and significantly increased the relative abundance of phylum *Verrucomicrobiota*. At the family level, KU treatment decreased the abundance of *Lachnospiraceae* while significantly enriching *Barnesiellaceae* and *Akkermansiaceae* in EAE mice (Figure 2C and Figure S3). These observations demonstrate that KU reshapes intestinal microbial community structure in EAE model mice.

**FIGURE 2:**
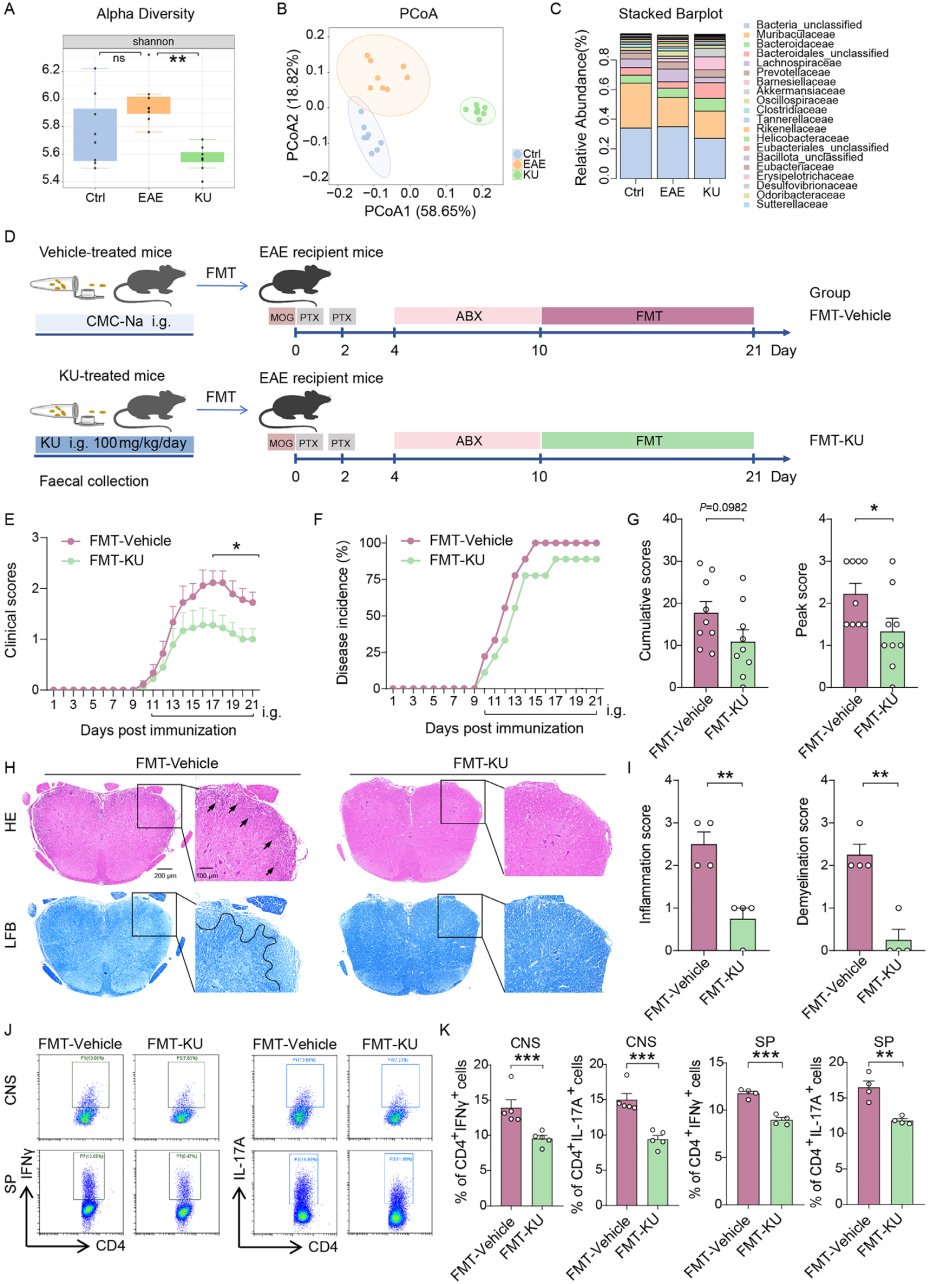
KU modulates gut microbiota to ameliorate EAE. (A) Alpha diversity (Shannon index) of gut microbiota in the Ctrl, vehicle‐treated EAE, and KU‐treated EAE groups (*n* = 8 per group). Pair‐wise comparisons were performed using Wilcoxon rank‐sum test. (B) Principal coordinate analysis (PCoA) of gut microbiota based on Bray‐Curtis distance (pseudo‐*F* = 30.12, *R* ^2^ = 0.741, *p* < 0.001); each dot represents an individual sample. (C) Stacked bar plot showing the relative abundance of gut microbiota at the family level in the Ctrl, vehicle‐treated EAE, and KU‐treated EAE groups. (D) Schematic timeline of ABX treatment and FMT and EAE induction: EAE mice received FMT from vehicle‐treated or KU‐treated donor mice (100 mg kg^−1^, intragastric). (E) Clinical scores of FMT‐Vehicle and FMT‐KU groups (*n =* 9 per group). (F) Disease incidence curves of EAE mice. (G) Statistical analysis of cumulative clinical scores and peak score. (H) Representative HE staining and LFB staining of spinal cord tissues from FMT‐Vehicle and FMT‐KU mice. (I) Inflammation score and demyelination score in the spinal cord (*n =* 4 per group). (J) Flow cytometric plots of CD4^+^IFNγ^+^ (Th1) and CD4^+^IL‐17A^+^ (Th17) cells in the CNS and SP. (K) Quantification of CD4^+^IFNγ^+^ and CD4^+^IL‐17A^+^ cell percentages in the CNS and SP (*n =* 5 per group). Data are presented as mean ± SEM. *p < 0.05, **p < 0.01, ***p < 0.001 vs. EAE mice that received FMT from vehicle‐treated donors.

To further clarify whether gut microbiota mediates the therapeutic effects of KU against EAE, we performed an FMT experiment. Fresh fecal supernatants from donor mice in the vehicle group or KU treatment group were orally transferred to the EAE recipient mice (Figure 2D). Compared with mice receiving microbiota from the vehicle‐treated group, mice receiving microbiota from the KU‐treated group had milder EAE disease severity, with significantly lower peak clinical scores (Figure 2E–G; *p* < 0.05) and cumulative scores that trended downward but were not statistically significant (Figure 2G; *p* = 0.0982). Histopathological analysis confirmed that transplantation of KU‐modified microbiota alleviated inflammatory infiltration and demyelination in the spinal cord of EAE mice (Figure 2H,I; *p <* 0.01). Flow cytometric analysis showed that the proportions of Th1 and Th17 cells in the CNS and spleen of EAE mice receiving KU‐modified microbiota was significantly lower than that of those receiving vehicle‐microbiota recipients (Figure 2J,K; *p <* 0.01 and *p <* 0.001). Collectively, these findings indicate that gut microbiota plays a crucial role in mediating KU's protective effects against EAE.

### 2.3 KU Enriches *Akkermansia Muciniphila* to Mitigate EAE

We performed metagenomic analysis to identify the top 10 differentially abundant microbial taxa across the control, vehicle‐treated EAE, and KU‐treated EAE groups (Figure 3A). Among these taxa, the relative abundance of *Akkermansia muciniphila* (*A. muciniphila*) was significantly elevated in the KU‐treated EAE group compared with the vehicle‐treated EAE group. This finding was further validated by LEfSe analysis (Figure 3B). To clarify the contribution of *A. muciniphila* to KU's protective effects against EAE, we conducted antibiotic depletion and bacterial supplementation experiments (Figure 3C). Following antibiotic‐mediated gut microbiota depletion, the therapeutic benefits of KU against EAE were completely abrogated. No improvements in clinical scores, CNS inflammatory infiltration and demyelination, and proportions of Th1 and Th17 cells were observed compared with the vehicle‐treated EAE group (Figure 3D,E; *p >* 0.05). Notably, *A. muciniphila* supplementation restored KU's therapeutic efficacy in antibiotics‐pretreated EAE mice, significantly reducing clinical scores, alleviating CNS pathological damage, and suppressing Th1 and Th17 cell proportions (Figure 3D–K; *p <* 0.05, *p <* 0.01, and *p <* 0.001). Furthermore, in vitro culture experiments demonstrated that KU could promote the proliferation of *A. muciniphila* (Figure S4; *p <* 0.001). Collectively, these data indicate that gut microbiota, especially *A. muciniphila*, is an indispensable mediator for KU's disease‐alleviating effects in EAE, and that its mechanism of action may be related to regulating Th1 and Th17 responses.

**FIGURE 3:**
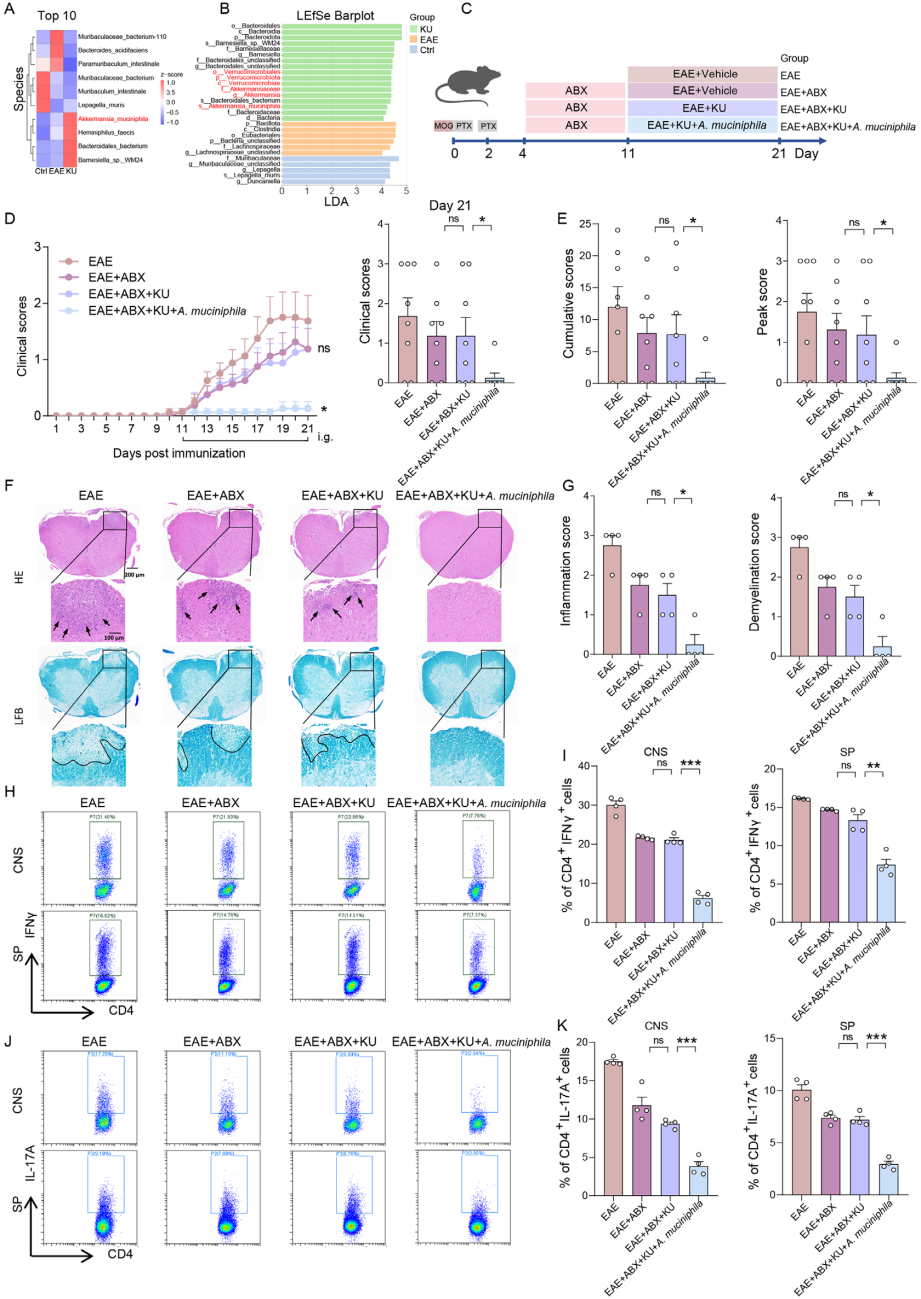
KU ameliorates EAE in an *A. muciniphila*‐dependent manner. (A) Heatmap showing the relative abundance of the top 10 gut microbial species in the Ctrl, vehicle‐treated EAE, and KU‐treated groups (*n =* 8 per group). (B) Linear discriminant analysis effect size (LEfSe) analysis was performed to screen differentially abundant microbial taxa across the three groups. Taxa with an LDA score > 3 were defined as potential microbial biomarkers with statistical significance. (C) Schematic timeline of ABX pretreatment, KU administration, *A. muciniphila* supplementation, and EAE induction. (D) Clinical scores of EAE, EAE+ABX, EAE+ABX+KU, and EAE+ABX+KU+*A. muciniphila* (*n =* 8 per group). (E) Cumulative clinical scores and peak score in each group. (F) Representative HE staining and LFB staining of the spinal cord. (G) Spinal cord inflammation score and demyelination score (*n =* 4 per group). (H) Flow cytometric plots of CD4^+^IFNγ^+^ (Th1) cells in the CNS and SP of each group. (I) Quantification of CD4^+^IFNγ^+^ cell percentages in the CNS and SP (*n =* 4 per group). (J) Flow cytometric plots of CD4^+^IL‐17A^+^ (Th17) cells in the CNS and SP of each group. (K) Quantification of CD4^+^IL‐17A^+^ cell percentages in the CNS and SP (*n =* 4 per group). Data are presented as mean ± SEM. *p < 0.05, **p < 0.01, ***p < 0.001; ns, *p >* 0.05.

### 2.4 *A. Muciniphila* Elevates the Tryptophan Metabolite Indole‐3‐Acetaldehyde

To further investigate the functional alterations in the gut microbiota following KU intervention, we performed untargeted metabolomics analysis on mouse fecal samples. Partial least squares discriminant analysis (PLS‐DA) revealed clear separation of fecal metabolic profiles among the Ctrl, EAE and KU groups (Figure 4A). KEGG pathway enrichment analysis indicated that tryptophan metabolism was ranked among the most significantly perturbed pathways after KU administration (Figure 4B). Further metagenomic functional annotation analysis revealed that *A. muciniphila* exerted prominent functional contributions to the tryptophan metabolic pathway (Figure 4C). Among the top 30 differentially abundant metabolites, we focused on indole‐3‐acetaldehyde (IAAld), a tryptophan metabolite generated through the indole biosynthetic pathway, and found that IAAld levels were markedly elevated upon KU treatment (Figure 4D,E). To further integrate microbial composition and functional phenotypes, we conducted metagenomic‐metabolomic correlation analysis and found that the abundance of *A. muciniphila* positively correlated with IAAld levels but negatively correlated with EAE clinical score, cumulative score, and peak score (Figure 4F). Then, to determine whether KU facilitates intestinal IAAld accumulation and subsequent brain penetration, as well as to verify the regulatory effect of *A. muciniphila* on IAAld production, we quantitatively detected IAAld levels using a semi‐quantitative LC‐MS/MS method (Figure S5). Low IAAld levels were observed in brain tissues of control and vehicle‐treated EAE mice, whereas KU treatment significantly elevated cerebral IAAld concentrations (Figure 4G; *p <* 0.01). Another independent cohort of EAE mice showed consistent results in vehicle‐ and KU‐treated mice (Figure S6). In addition, KU treatment‐induced *A. muciniphila* enrichment was positively correlated with IAAld levels in serum and brain tissues, and negatively correlated with EAE clinical scores.

**FIGURE 4:**
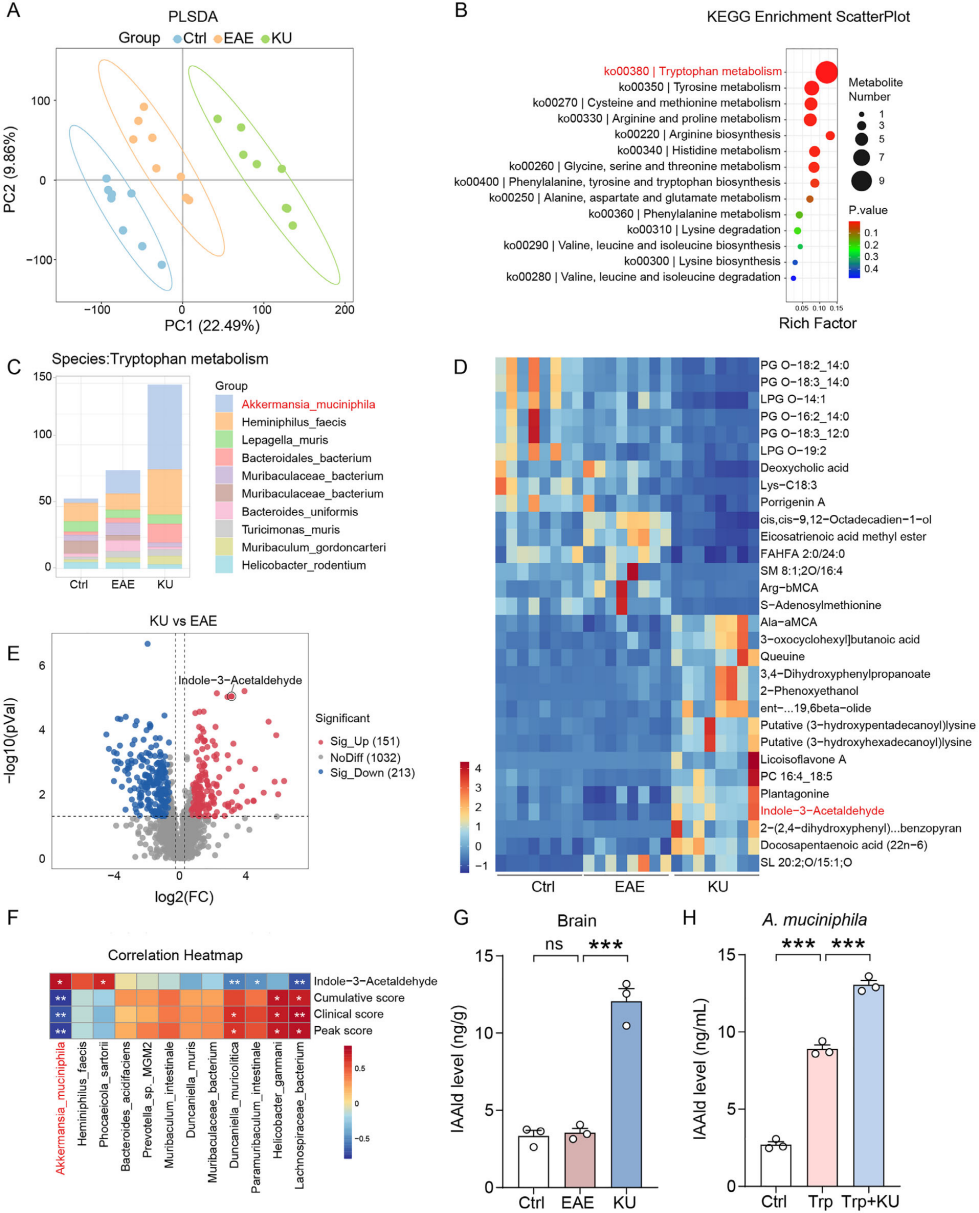
KU upregulates tryptophan‐derived IAAld that is strongly linked to *A. muciniphila*. (A) Partial least squares discriminant analysis (PLS‐DA) score plot of metabolomic profiles in the Ctrl, vehicle‐treated EAE, and KU‐treated EAE groups (*n =* 8 per group). (B) KEGG pathway enrichment scatter plot of differential metabolites. (C) Bar plot illustrating the contribution of gut bacterial species to tryptophan metabolism in the Ctrl, EAE, and KU groups. (D) Heatmap of differential metabolites in the Ctrl, vehicle‐treated EAE, and KU‐treated EAE groups. (E) Volcano plot of differential metabolites between KU‐treated EAE and vehicle‐treated EAE groups. Red dots represent significantly upregulated metabolites (Sig_Up, 151), blue dots represent significantly downregulated metabolites (Sig_Down, 213), and gray dots represent non‐differential metabolites (NoDiff). (F) Spearman rank‐order correlation heatmap showing correlations between representative gut microbial species, the metabolite indole‐3‐acetaldehyde, and EAE clinical scores. Color gradient represents Spearman correlation coefficient. (G) The content of IAAld in the brain tissues of mice in each group (*n =* 3 per group). (H) Detection of IAAld content in the culture supernatant of *A. muciniphila* after supplementation with tryptophan (0.2 g L^−1^) and KU (10 µM), with 24 h anaerobic incubation at 37°C (*n =* 3 per group). Data are presented as mean ± SEM. *p < 0.05, **p < 0.01, ***p < 0.001; ns, *p >* 0.05.

To establish *A. muciniphila*’s role in IAAld production, we performed in vitro and in vivo experiments. In vitro, we detected trace IAAld in the *A. muciniphila* culture supernatant. Upon tryptophan supplementation, IAAld levels increased significantly, and KU treatment further enhanced *A. muciniphila*‐driven tryptophan catabolism to generate IAAld (Figure 4H; *p <* 0.05 and *p <* 0.001). We further performed antibiotic‑driven gut microbiota depletion followed by *A. muciniphila* gavage in EAE mice. Compared with the EAE+ABX group, *A. muciniphila* supplementation substantially ameliorated EAE clinical severity (Figure S7A). Consistently, *A. muciniphila* supplementation markedly boosted IAAld levels in feces, serum, and brain tissue (Figure S7B). Spearman correlation analysis further showed that IAAld levels in feces, serum, and brain were all negatively correlated with EAE clinical scores (Figure S7C; *P* < 0.05), reinforcing the link between *A. muciniphila*‑derived IAAld and disease improvement. Collectively, these results show that *A. muciniphila* elevates the tryptophan metabolite IAAld to ameliorate EAE, which may account for KU's protective effects against EAE.

### 2.5 IAAld Suppresses Th1 and Th17 Cell Proportions and Alleviates EAE

To clarify the potential effect of IAAld on EAE, EAE mice were given oral IAAld starting from the day of EAE onset. First, we confirmed that oral IAAld treatment markedly increased IAAld levels in serum and brain tissue (Figure S8; *p* < 0.01, *p* < 0.001). Compared with the vehicle‐treated EAE group, IAAld treatment mitigated EAE disease severity and incidence (Figure 5A–C; *p* < 0.05 and *p* < 0.001). Specifically, IAAld significantly lowered peak clinical scores (Figure 5D; *p* < 0.05), and cumulative scores showed a downward trend but were not statistically significant (Figure 5D; *p* = 0.0535). Histopathological analysis further confirmed that IAAld significantly alleviated inflammatory infiltration and demyelination in the spinal cord (Figure 5E,F; *p <* 0.01). Meanwhile, IAAld significantly reduced Th1 and Th17 cell proportions in the CNS and spleen (Figure 5G,H; *p <* 0.05 and *p <* 0.001). Collectively, these findings suggest that tryptophan‐derived IAAld serves as a key downstream effector molecule of *A. muciniphila* and plays a critical role in KU‐mediated disease‐alleviating effects in EAE.

**FIGURE 5:**
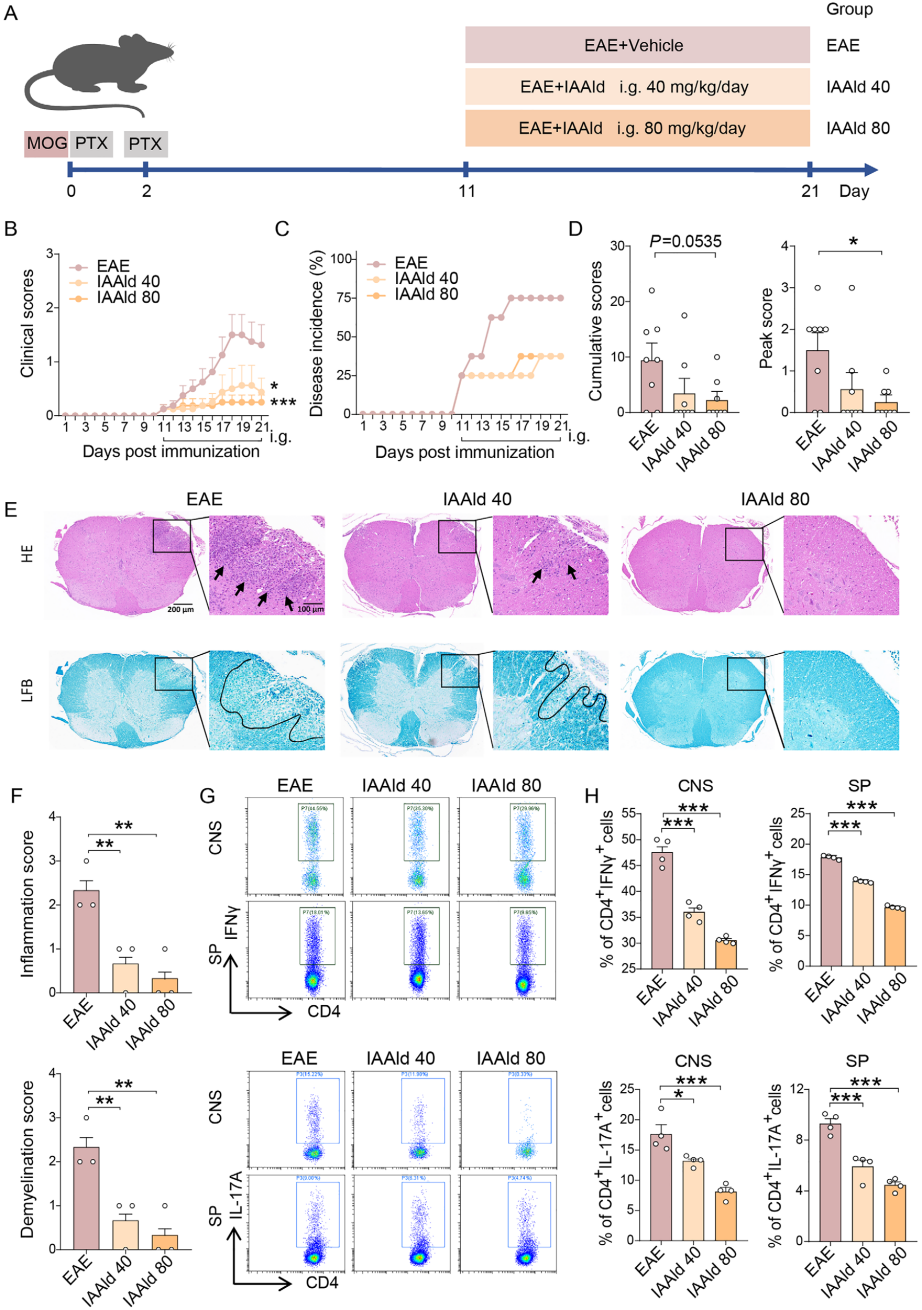
IAAld alleviates EAE and reduces Th1 and Th17 cell proportions. (A) Schematic timeline of EAE induction and IAAld administration (*n =* 8 per group). (B) Clinical scores of vehicle‐treated EAE, EAE+IAAld 40 mg/kg/day (IAAld 40), and EAE+IAAld 80 mg/kg/day (IAAld 80) groups. (C) Disease incidence curves of EAE mice with different doses of IAAld treatment. (D) Quantification of cumulative clinical scores and peak score in each group. (E) Representative HE staining and LFB staining of spinal cord tissues from the vehicle‐treated EAE, IAAld 40, and IAAld 80 groups. (F) Spinal cord inflammation score and demyelination score (*n =* 3 per group). (G) Flow cytometric plots of CD4^+^IFNγ^+^ (Th1) and CD4^+^IL‐17A^+^ (Th17) cells in the CNS and SP of each group. (H) Quantification of CD4^+^IFNγ^+^ and CD4^+^IL‐17A^+^ cell percentages in the CNS and SP (*n =* 4 per group). Data are presented as mean ± SEM. *p < 0.05, **p < 0.01, ***p < 0.001.

### 2.6 IAAld Inhibits Th1 and Th17 Cell Differentiation via the AhR/JAK/STAT Pathway

By adopting a network pharmacology strategy, we performed intersection mapping between IAAld targets and MS disease targets and combined it with KEGG pathway enrichment analysis. The results showed that the pathways related to Th1 and Th17 cell differentiation were highly enriched (Figure 6A). Considering that indole derivatives are classic AhR ligands [26, 27], we then performed molecular docking as an in silico prediction to evaluate the binding interactions between IAAld and AhR. The results confirmed that both molecules have binding potential, with a binding energy of −6.6 kcal mol^−1^ (Figure 6B). We subsequently performed AhR knockdown with siRNA to explore the role of AhR in mediating IAAld's effects on Th1 and Th17 differentiation in vitro. Specifically, IAAld suppressed Th1 cell differentiation by upregulating AhR protein expression and promoting AhR nuclear translocation, whereas AhR knockdown reversed these effects (Figure 6C–E). We observed similar regulatory effects of IAAld and AhR knockdown under Th17 differentiation conditions (Figures 6C–E). Given that the JAK/STAT signaling pathway is closely related to Th1 and Th17 cell differentiation, we further investigated the underlying molecular mechanisms. Specifically, we observed that IAAld inhibited JAK2 phosphorylation, upregulated the negative regulatory proteins SOCS1 and SOCS3, and further suppressed STAT4 and STAT3 phosphorylation. All these changes were reversed by targeted AhR siRNA knockdown (Figure 6F,G). Collectively, our findings indicate that AhR mediates IAAld's inhibitory effects on Th1 and Th17 differentiation by suppressing JAK/STAT signaling.

**FIGURE 6:**
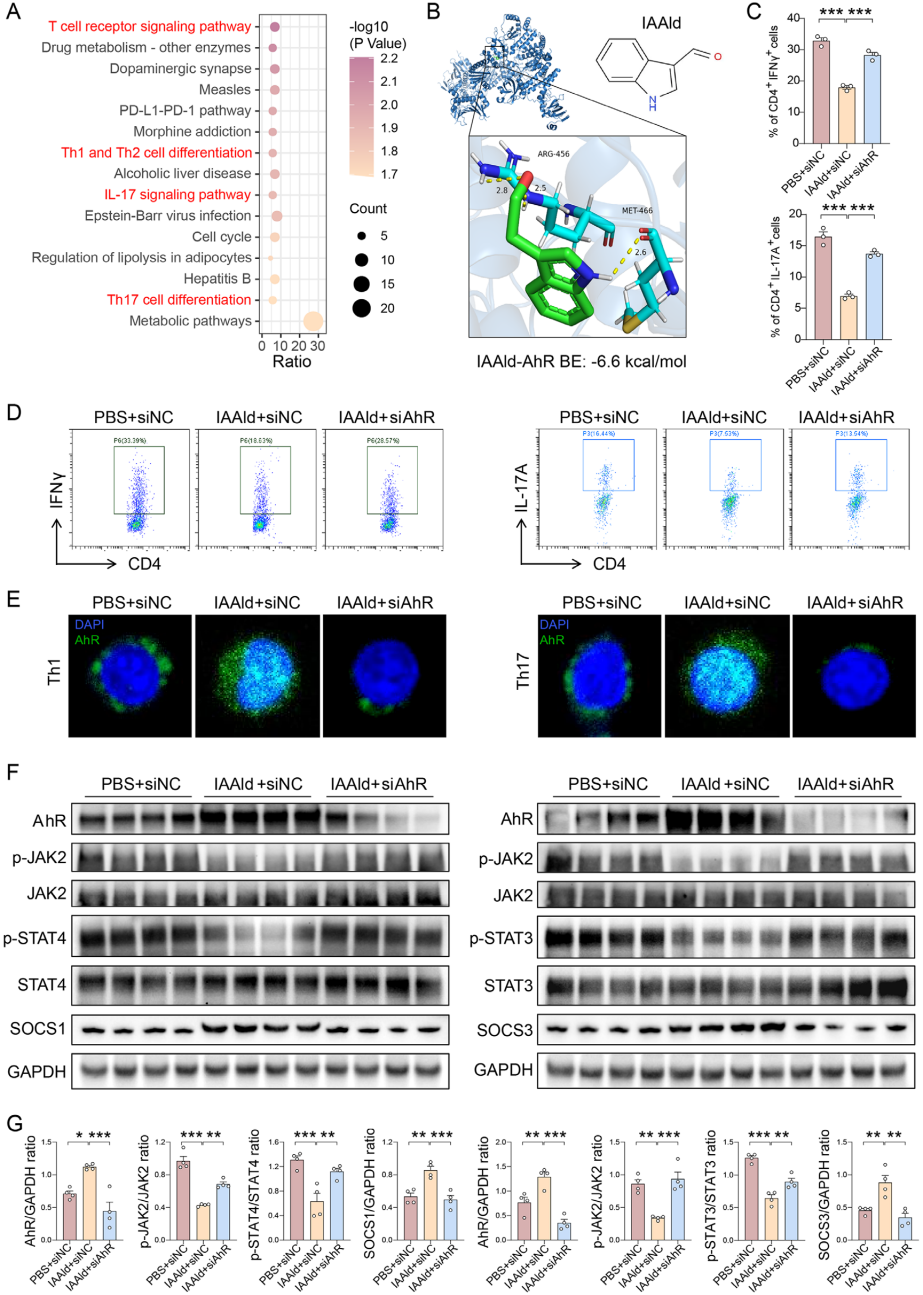
IAAld suppresses Th1 and Th17 cell differentiation in an AhR‐dependent manner by repressing JAK/STAT signaling. (A) KEGG pathway enrichment analysis of the intersectional targets of IAAld and MS. (B) In silico prediction of the binding interaction between IAAld and AhR by molecular docking. The calculated binding energy (BE) is −6.6 kcal/mol (inset: chemical structure of IAAld). (C,D) Flow cytometric quantification and representative plots of CD4^+^IFNγ^+^ (Th1) and CD4^+^IL‑17A^+^ (Th17) cells under in vitro differentiation conditions across PBS, IAAld (10 µM), and IAAld+siAhR (AhR knockdown by siRNA) groups (*n =* 3 per group). (E) Immunofluorescence staining of AhR (green) in Th1 and Th17 cells of each group, and cell nuclei were stained with DAPI (blue). (F) Western blot analysis showing protein levels of AhR, p‑JAK2, JAK2, p‑STAT4, STAT4, p‑STAT3, STAT3, SOCS1, SOCS3, and GAPDH in Th1 (left) and Th17 (right) cells. (G) Quantitative analysis of AhR/GAPDH ratio, p‐JAK2/JAK2 ratio, p‐STAT4/STAT4 ratio, p‐STAT3/STAT3 ratio, SOCS1/GAPDH ratio and SOCS3/GAPDH ratio in each group (*n =* 4 per group). Data are presented as mean ± SEM. *p < 0.05, **p < 0.01, ***p < 0.001.

### 2.7 IAAld Treatment Mitigates EAE in an AhR‐Dependent Manner

Based on the results of in vitro experiments, we further explored whether IAAld has a disease‐alleviating effect in EAE mice. The qPCR analysis showed that IAAld dose‐dependently up‐regulated the mRNA expression of *Ahr* and its downstream target genes *Cyp1a1* and *Cyp1b1* in the spinal cord of EAE mice (Figure 7A; *p <* 0.05, *p <* 0.01, *p <* 0.001), indicating that IAAld activates the AhR signaling pathway. Next, we conducted in vivo experiments with AhR‐specific inhibitor (AhRi, StemRegenin 1, SR1) (Figure 7B). Compared with the vehicle‐treated EAE group, IAAld significantly mitigated EAE (Figure 7C; *p <* 0.001), showing decreased incidence (50% vs 100%), cumulative and peak scores. In contrast, administration of AhRi significantly reversed IAAld's protective effect (Figure 7C–E; *p <* 0.01, *p <* 0.001). Consistent with these findings, histopathological analysis showed that IAAld alleviated inflammatory infiltration and demyelination in the spinal cords of EAE mice, whereas AhRi reversed this effect (Figure 7F,G; *p <* 0.05, *p <* 0.01). Flow cytometry showed that the significantly reduced proportions of Th1 (CD4^+^IFNγ^+^) and Th17 (CD4^+^IL‐17A^+^) cells in IAAld‐treated EAE mice were restored by AhRi administration (Figure 7H,I; *p <* 0.05, *p <* 0.01, *p <* 0.001). All the findings support the critical role of AhR signaling in mediating IAAld's efficacy and indicate that IAAld mitigates EAE in an AhR‐dependent manner.

**FIGURE 7:**
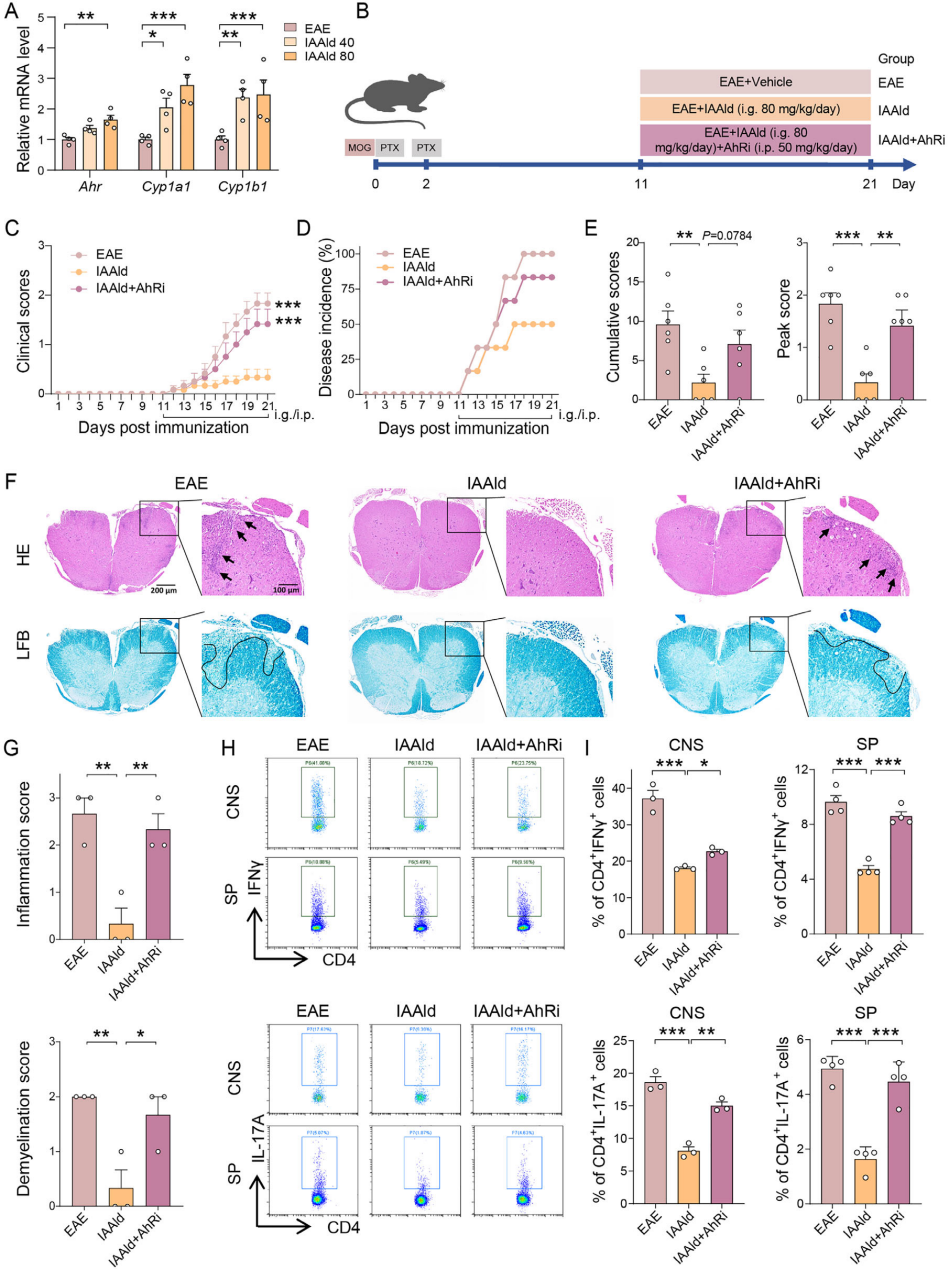
IAAld treatment mitigates EAE in an AhR‐dependent manner. (A) Relative mRNA expression levels of *Ahr, Cyp1a1, and Cyp1b1* in the spinal cord of EAE mice treated with vehicle, IAAld 40, or IAAld 80 groups. (B) Schematic timeline depicting the experimental protocol for EAE induction, followed by administration of IAAld (80 mg/kg/day) and AhR inhibitor (AhRi; SR1, 50 mg/kg/day). (C) Clinical scores for EAE mice treated with vehicle, IAAld, and IAAld+AhRi groups (*n* = 6 per group). (D) Disease incidence curves for EAE mice over time. (E) Quantification of cumulative clinical scores and peak score for each group. (F) Representative HE and LFB staining of spinal cord tissues for assessment of inflammation and demyelination, respectively. (G) Quantification of spinal cord inflammation and demyelination scores (*n =* 3 per group). (H) Representative flow cytometry plots indicating the percentages of CD4^+^IFNγ^+^ (Th1) and CD4^+^IL‐17A^+^ (Th17) cells in the CNS and SP of each group. (I) Quantification of CD4^+^IFNγ^+^ and CD4^+^IL‐17A^+^ cell percentages in the CNS and SP across groups (*n =* 3–4 per group). Data are presented as mean ± SEM. *p < 0.05, **p < 0.01, ***p < 0.001.

Considering that IAAld is the downstream effector molecule of KU, we next explored the role of AhR signaling in KU's therapeutic effects in EAE mice. As shown in Figure S9, SR1‐mediated AhR inhibition abrogated KU's disease‐alleviating effects in EAE, supporting IAAld‐driven AhR activation as an essential mechanism that limits pathogenic Th1/Th17 responses and neuroinflammation, and underscoring KU's therapeutic benefit in EAE.

## 3 Discussion

MS, a leading cause of non‐traumatic disability in young and middle‐aged adults, is closely related to the imbalance of CD4^+^ T cell subsets, especially the pro‐inflammatory Th1 and Th17 cell activation and the related cytokine secretion, which are recognized as the core mechanism driving CNS damage [28, 29]. Accordingly, inhibiting Th1 and Th17 cell differentiation and restoring immune homeostasis have become major strategies for MS treatment, and identifying candidate drugs that can suppress Th1 and Th17 responses is of great significance. In this study, we verified that KU, a major component of the traditional Chinese medicine *Radix Sophora Flavescens*, significantly alleviated disease severity and reduced CNS inflammatory infiltration and demyelination in EAE mice, primarily by reducing Th1 and Th17 cell proportions and responses. More strikingly, a novel mechanism related to gut microbiota‐metabolite‐immune axis has been demonstrated, for the first time, to mediate the disease‐alleviating effects of KU in EAE, thereby providing new insights into MS treatment.

*A. muciniphila* is one of the commensal gut bacterium and has been proven to play key roles in antagonizing obesity, improving type 2 diabetes, and delaying the aging process [30, 31]. However, the association between *A. muciniphila* and MS remains controversial. On the one hand, some researchers observed that the relative abundance of *A. muciniphila* in the intestines of MS patients was significantly higher than in healthy controls and speculated that it played a pro‐inflammatory role, contributing to an imbalance in the gut microbiota and immune‐mediated diseases such as MS [32, 33, 34, 35]. On the other hand, more studies have suggested that enrichment of *A. muciniphila* in MS patients may be a compensatory beneficial response, since *A. muciniphila* supplementation produced a clear disease‐alleviating effect in EAE mice. It may reshape gut microbiome homeostasis and induce anti‐inflammatory immune responses, thereby attenuating CNS damage [36, 37, 38, 39, 40]. In‐depth mechanistic studies are needed to clarify whether *A. muciniphila* exhibits diverse biological properties during the disease process of MS. Consistent with prior literature involving MS patients [41], we observed a mild increase in the relative abundance of *A. muciniphila* in EAE mice. Moreover, KU further enriched *A. muciniphila* in EAE mice and directly promoted its proliferation in vitro. Therefore, reduced disease severity in KU‐treated EAE mice is probably attributed to *A. muciniphila* driven by KU. The FMT experiment further verified this conclusion: transplanting fecal microbiota from KU‐treated donor mice effectively alleviated the disease severity in recipient EAE mice. Notably, when antibiotics were used to deplete the endogenous gut microbiota, KU's disease‐alleviating effects disappeared. However, KU's therapeutic effects could be restored when combined with *A. muciniphila* supplementation. Based on the evidence, *A. muciniphila* is identified as the key microbiota target for KU.

Tryptophan metabolism mainly occurs in the intestine and branches into three major metabolic pathways: indole, kynurenine, and serotonin [42]. Among them, the indole metabolic pathway is a key hub that mediates crosstalk between the gut microbiota and host immune and metabolic homeostasis. The gut microbiota is the major producer of metabolites from this pathway [43]. In this study, through multi‐omics analysis and experimental validation, we confirmed that KU markedly increased IAAld levels, a product of the indole metabolic pathway, in the feces, serum, and brain of EAE mice, and similar results were obtained when KU‐driven *A. muciniphila* was administered. These findings support IAAld as the effector molecule by which KU exerts its efficacy. Existing evidence indicates that indole and its derivatives are classical AhR ligands [19, 44, 45]. Previous studies have shown that the tryptophan metabolite indole‐3‐lactic acid can activate the AhR‐mediated pathway to inhibit STAT3 phosphorylation and protect from intestinal vascular barrier dysfunction due to intestinal ischemic injury [46]; AhR activation alleviates damp‐heat colitis by regulating the STAT signaling pathway, and the latter plays an indispensable role in promoting Th1 and Th17 differentiation [47]. Therefore, we hypothesized that the tryptophan metabolite‐AhR‐immune axis may serve as the potential mechanism responsible for KU's efficacy since tryptophan‐derived IAAld has been identified as the key downstream molecule of KU in our study. As expected, we observed that IAAld suppressed Th1 and Th17 differentiation by activating AhR and promoting AhR nuclear translocation. Mechanistically, IAAld upregulates AhR expression, inhibits JAK2, STAT4, and STAT3 phosphorylation, and promotes expression of the negative regulatory proteins SOCS1 and SOCS3. These changes can be reversed by AhR knockdown via siRNA. Strikingly, AhR inhibition with SR1 treatment further demonstrates AhR's essential role in ameliorating EAE disease severity in the context of IAAld or KU treatment. These findings support our hypothesis that the IAAld‐AhR‐immune axis underscores KU's efficacy in EAE.

Apart from KU's anti‐inflammatory and immunoregulatory roles [20, 21, 48], our study, for the first time, extends its action to the field of gut microbiota‐metabolite modulation. One previous study has reported that in vitro naive CD4^+^ T cells exposed to KU exhibited inhibited Th1 and Th17 cell differentiation, indicating the direct effect of KU on T cells [24]. However, this cannot fully cover the mechanism of action of KU when administered orally in EAE mice. As a natural flavonoid, KU has been reported to exhibit relatively low intestinal absorption and bioavailability, thereby limiting its direct effects on peripheral or target organ‐infiltrating T cells [49]. Therefore, we hypothesize that other modes of action, such as relying on intermediate effector factors, may mediate the pharmacological effects of KU in vivo. Here, we focused on the gut microbiota and metabolites and demonstrated that KU decreased the proportions of Th1 and Th17 cell differentiation in CNS and spleen tissues through *A. muciniphila*‐derived IAAld and the downstream AhR signaling pathways. This finding further validates the contribution of gut microbiota and metabolites to the in vivo effects of KU and enrich our understanding of KU‐mediated regulation of T cell differentiation. Moreover, our results support developing novel treatment strategies, such as gut microbiota engineering, targeted delivery of microbial metabolites, and AhR‐specific agonist development, for MS treatment and prevention.

However, this study still has several limitations. First, our research focused only on the EAE animal model, and translating the key findings to MS patients requires further clinical verification. Second, the specific molecular mechanisms by which *A. muciniphila* modulates tryptophan metabolism and KU promotes *A. muciniphila* proliferation and enrichment remain to be elucidated and require in‐depth exploration. Third, an IAAld‐neutralizing strategy is not technically feasible because well‐validated neutralizing antibodies or specific chemical inhibitors targeting IAAld are lacking, which hinders the implementation of a “rescue” experiment focusing on IAAld function in vivo. Finally, we investigated the effects of KU over a 21‐day time course in this study, and further investigation is needed to validate its long‐term efficacy and safety.

In conclusion, this study confirms for the first time that gut microbiota‐derived IAAld contributes to the disease‐alleviating effects of KU in EAE (Figure 8). Briefly, KU‐enriched *A. muciniphila* promotes the tryptophan metabolite IAAld production, and the latter in turn activates AhR and promotes its nuclear translocation. Activated AhR suppresses JAK2 phosphorylation, promotes SOCS1 and SOCS3 expression, and further reduces the phosphorylation of STAT4 and STAT3. This cascade consequently constrains Th1 and Th17 cell differentiation and alleviates EAE. Beyond defining a previously unappreciated mechanism underlying KU‐mediated EAE amelioration, our research establishes an interaction network centered on the gut microbiota‐derived IAAld‐AhR‐immune axis, offering candidate therapeutic targets for MS and other autoimmune disorders.

**FIGURE 8:**
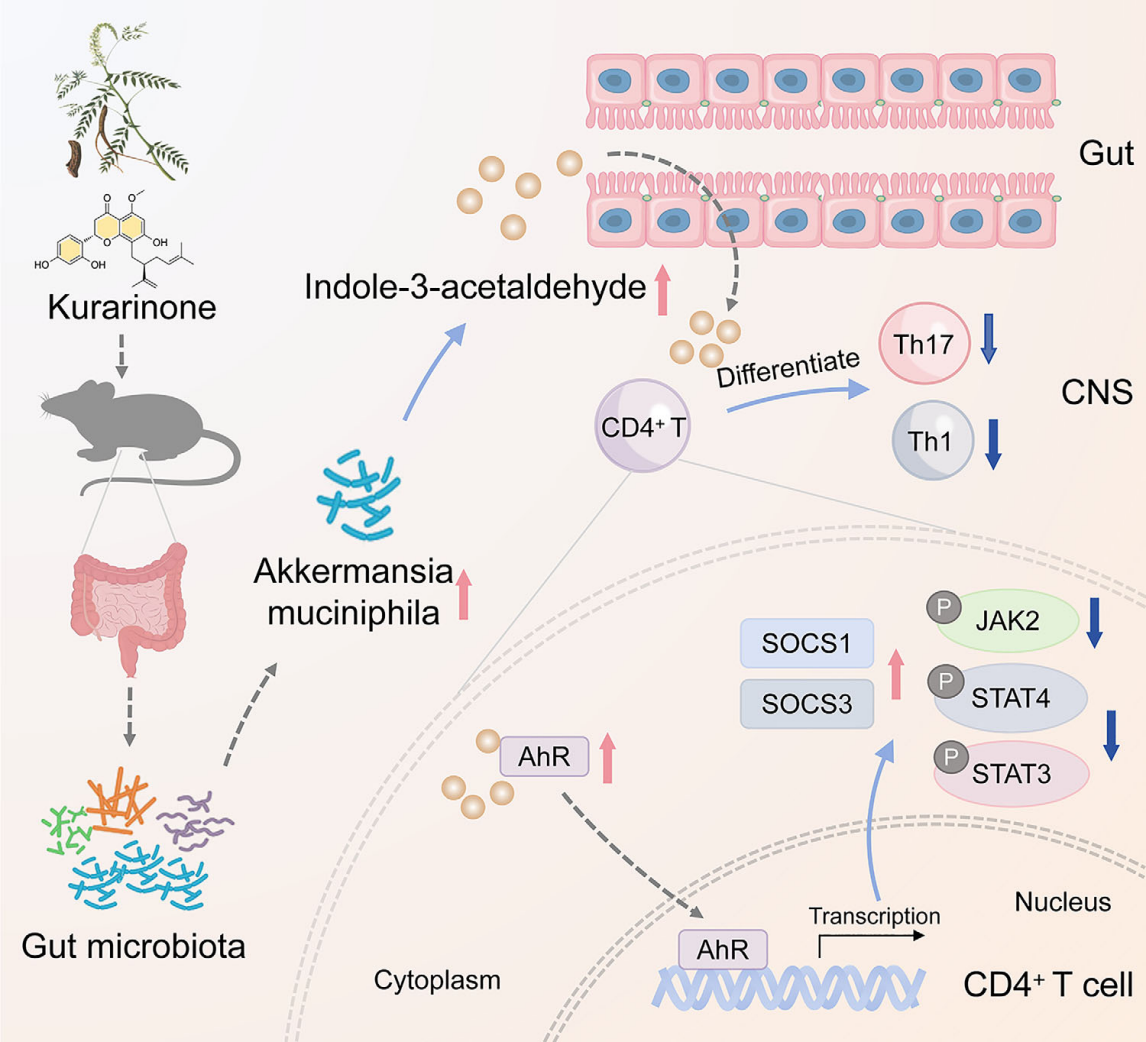
Graphical abstract. Schematic diagram illustrating the molecular mechanism by which KU ameliorates EAE through the gut microbiota‐IAAld‐AhR axis. KU reshapes gut microbiota composition in EAE mice, specifically increasing the abundance of *Akkermansia muciniphila*. *A. muciniphila* facilitates intestinal production of indole‐3‐acetaldehyde (IAAld) and the latter crosses the intestinal barrier to enter peripheral circulation and CNS, where IAAld activates AhR and promotes its nuclear translocation in CD4^+^ T cells. Once in the nucleus, activated AhR upregulates SOCS1 and SOCS3 expression and suppresses phosphorylation of JAK2 and its downstream targets, STAT4 and STAT3. This cascade restrains CD4^+^ T cell differentiation into Th1 and Th17 subsets, ultimately mitigating CNS inflammation and demyelination in EAE mice.

## 4 Materials and Methods

### 4.1 Reagents

Kurarinone was obtained from Chengdu Alfa Biotechnology Co., Ltd. (CAT: 34981‐26‐5). Metronidazole (CAT: MB2200‐1), Vancomycin (CAT: MB1260), Neomycin (CAT: MB1716), and Ampicillin (CAT: MB1507) were purchased from Meilunbio. Indole‐3‐acetaldehyde (IAAld) was purchased from Shanghai Acmec Biochemical Technology Co., Ltd (CAT: 2591‐98‐2). RPMI 1640 medium and fetal bovine serum (FBS) were purchased from Gibco. Myelin oligodendrocyte glycoprotein_35‐55_ (MOG_35‐55_) peptide was obtained from ChinaPeptides. Pertussis toxin (PTX) was purchased from List Biological Laboratories (CAT: 181). Freund's incomplete adjuvant was purchased from Sigma‐Aldrich (CAT: F5506). *Mycobacterium tuberculosis* was purchased from BD Bioscience (CAT: MBS231141). Cell stimulation cocktail (CAT: 00‐4970‐03), Fixation buffer (CAT: 88‐8824‐00), Fluorescent‐labeled anti‐CD4 (CAT: 11‐0041‐82), anti‐IL‐17A (CAT: 16‐9017‐82), and anti‐IFNγ (CAT: 12‐7319‐42) were obtained from eBioscience. Anti‐mouse IFNγ (CAT: 517901), IL‐4 (CAT: 504101), CD3s (CAT: 1003015), and CD28 (CAT: 1021015) antibodies were obtained from BioLegend. Recombinant TGFβ (CAT: 100–21), IL‐1β (CAT: 211‐11), IL‐6 (CAT: 216‐16), IL‐23 (CAT: 200–23), and IL‐2 (CAT: 212‐12) proteins were obtained from PeproTech. Percoll was obtained from Cytiva (CAT: 17089101). *Akkermansia muciniphila* strains were purchased from BNCC (CAT: BNCC341917).

### 4.2 Animals

Female C57BL/6 mice aged 6–8 weeks were purchased from GemPharmatech and maintained in the Laboratory Animal Center of Fourth Military Medical University. All mice were maintained under SPF conditions and housed under a 12:12‐h light‐dark cycle, with humidity of 40%–70%, ambient temperature of 26°C, and sufficient food and water. Mice were group‐housed with cage rotation for 2 weeks before the experiments. All animal experimental procedures were approved by the Institutional Animal Care and Use Committee of Fourth Military Medical University.

### 4.3 EAE Induction and Treatment

The mice were inoculated with an emulsion comprising incomplete Freund's adjuvant, MOG_35‐55_ (200 µg) and H37RA (500 µg) at 3 subcutaneous locations on the dorsal surface of the mice. The mice were intraperitoneally injected with 200 ng of pertussis toxin (PTX) on the induction day and 48 h later. EAE clinical scores were assessed daily: 0 (no symptoms), 1 (tail limpness), 2 (hindlimb weakness), 3 (hindlimb paralysis), 4 (tetraplegia), and 5 (moribund/death). On 11 dpi, the KU group was orally administered 100 mg kg^−1^ of KU treatment (*n* = 8 per group), the IAAld group was orally administered 40 or 80 mg kg^−1^ of IAAld treatment (*n* = 8 per group), the IAAld+AhRi group was orally administered 80 mg kg^−1^ IAAld and intraperitoneally injected with 50 mg kg^−1^ SR1 (*n* = 6 per group), and the EAE group received PBS until 21 dpi.

### 4.4 Histopathological Assessment

The mice were anesthetized with a 10% urethane solution, then perfused with normal saline and fixed in 4% paraformaldehyde. The spinal cord tissues were removed, embedded in paraffin, and cut into 3 µm sections. Hematoxylin and Eosin and Luxol Fast Blue (LFB) staining were performed according to previously described methods [50]. Inflammatory infiltration and demyelination were assessed as previously reported [51]: 0, none; 1, a few inflammatory cells/punctate demyelination; 2, local inflammatory infiltration around the blood vessel/a few areas of demyelination; and 3, perivascular inflammatory cells spread extensively into adjacent tissues/extensive demyelination.

### 4.5 Real‐Time Quantitative PCR (RT‐qPCR)

The spinal cord tissues were collected on 21 dpi. Total RNA was extracted using the Trizol (Coolaber, RE600) method. Total RNA was reverse‐transcribed into cDNA using the PrimerScript RT reagent kit (Takara, RR047A) following the manufacturer's instructions. qPCR was performed using SYBR Green Master Mix (Deeyeebio, DY20306) on the ABI ViiA7 instrument (Applied Biosystems). The mRNA expression level were normalized to *gapdh*. The primer sequences used were: *gapdh* forward, 5’‐ATGTGTCCGTCGTGGATCTGA‐3’, *gapdh* reverse, 5’‐ATGCCTGCTTCACCACCTTCT‐3’; *T‐bet* forward: 5’‐AGCAAGGACGGCGAATGTT‐3’; *T‐bet* reverse: 5’‐GGGTGGACATATAAGCGGTTC‐3’; *RORγt* forward: 5’‐GACCCACACCTCACAAATTGA‐3’; *RORγt* reverse: 5’‐AGTAGGCCACATTACACTGCT‐3’; *Cyp1a1* forward: 5’‐GAGGCTAAACAGCCTTCCCA‐3’; *Cyp1a1* reverse: 5’‐ACAGGAGCACCCTGTTTGTT‐3’; *Cyp1b1* forward: 5’‐CCAGATCCCGCTGCTCTACA‐3’; *Cyp1b1* reverse: 5’‐TGGACTGTCTGCACTAAGGCTG‐3’; *Ahr* forward: 5’‐CGGCTTCTTGCAAAACACAGT‐3’; *Ahr* reverse: 5’‐GTAAATGCTCTCGTCCTTCTTCATC‐3’.

### 4.6 Western Blot

SDS loading buffer (Deeyeebio, 50053) was added to the cell lysis solution, and the proteins (30 µg/lane) were separated by SDS‐PAGE and transferred onto the PVDF membrane, and probed with primary antibodies against phospho‐JAK2 (Tyr1007/1008) (CST, 3771), JAK2 (CST, 3230), phospho‐STAT3 (Ser705) (CST, 9145), phospho‐STAT4 (Tyr693) (CST, 4134), STAT3 (Proteintech, 10253‐2‐ap), STAT4 (CST, 2653), SOCS1 (CST, 55313), SOCS3 (Proteintech, 14025‐1‐ap), and AhR (Proteintech, 67785‐1‐lg), followed by HRP‐conjugated secondary antibodies (Proteintech, SA00001‐1, SA00001‐2). Signals were detected via ECL (Deeyeebio, 30208), with GAPDH (Proteintech, 60004‐1‐Ig) as the loading control.

### 4.7 Flow Cytometric Analysis

For in vivo experiments, CNS mononuclear cells (MNCs) were isolated by grinding and separating mouse brain and spinal cord tissues; spleen cells were obtained by grinding spleen tissue; detailed methods are provided below. Mouse brain and spinal cord tissues were isolated and homogenized with the inner tube of a 2 mL syringe, then resuspended in 10 mL of 30% Percoll solution and filtered through a 100 µm nylon cell strainer. The filtrate was layered onto 70% Percoll solution and centrifuged, and the single‐cell suspension was collected from the intermediate layer. The suspension was then centrifuged at 800 g for 10 min. The cell precipitates were CNS MNCs. After grinding the spleen tissue into single cells, they were incubated with red blood cell lysate for 5 min and then centrifuged to collect splenocytes. For in vitro experiments, naive CD4^+^ T cells were extracted from the splenocytes using the Mouse Naive CD4^+^ T Cell Isolation Kit (Thermo Fisher, 8804‐6824‐74). Anti‐CD3 (5 µg mL^−1^) was used to coat 48‐well cell culture plates in a 37°C incubator. The cells were incubated with anti‐CD28 (2 µg mL^−1^) under Th1 or Th17 differentiation conditions for 72 h. For Th1 differentiation, IL‐12 (20 ng mL^−1^) and anti‐mouse IL‐4 (10 µg mL^−1^) were added to stimulate cell differentiation. For Th17 differentiation, anti‐mouse IL‐4 (10 µg mL^−1^), anti‐mouse IFNγ (10 µg mL^−1^), IL‐1β (10 ng mL^−1^), IL‐6 (30 ng mL^−1^), TGF‐β1 (5 ng mL^−1^), IL‐2 (10 ng mL^−1^), and IL‐23 (30 ng mL^−1^) were added.

For gene knockdown experiments, purified naive CD4^+^ T cells were first pre‑activated with plate‑coated anti‑CD3 (5 µg mL^−1^) and soluble anti‑CD28 (2 µg mL^−1^) in complete medium containing IL‑2 (10 ng mL^−1^) for 18 h at 37°C. After pre‑activation without polarizing cytokines, 2 × 10^6^ activated CD4^+^ T cells per reaction were nucleofected with AhR siRNA or negative‑control siRNA using the Mouse T Cell Nucleofector Kit (Lonza, VPA‑1006) via program X‑001 on the Amaxa Nucleofector II device. The final siRNA concentration was 100 nM. After 2 h of recovery in fresh complete medium, the nucleofected cells were cultured under the Th1 or Th17 differentiation conditions for 72 h.

For intracellular staining, cells were stimulated with cell stimulation cocktail (plus protein transport inhibitors) (Thermo Fisher, 00‐4975‐93) for 4 h. Cell surface staining was first performed with FITC‐conjugated anti‐CD4 antibody at 4°C for 30 min in the dark. After washing, cells were fixed and permeabilized using a commercial intracellular staining kit according to the manufacturer's instructions. Subsequently, intracellular cytokine staining was carried out using PE‐conjugated anti‐IFNγ and APC‐conjugated anti‐IL‐17A antibodies to detect Th1 and Th17 cell populations, respectively. Th1 (CD4^+^ IFNγ^+^) and Th17 cells (CD4^+^ IL‐17A^+^) were quantified on Beckman BH16090.

### 4.8 Enzyme‐Linked Immunosorbent Assay (ELISA)

The mouse serum was detected using the IFNγ and IL‐17A ELISA kits (Elabscience, E‐EL‐M0047, E‐EL‐M0048) according to the kit instructions.

### 4.9 Fecal Bacteria DNA Sequencing and Bioinformatic Analysis

We extracted DNA using the following procedures. Briefly, DNA from different samples was extracted using the E.Z.N.A. Stool DNA Kit (D4015‐02, Omega) according to the manufacturer's instructions. Sample blanks consisting of unused swabs were processed alongside samples during DNA extraction and confirmed to yield no DNA amplicons. Total DNA was eluted in 50 µL of Elution buffer following the manufacturer‑modified protocol and stored at −80°C prior to PCR measurement, which was performed by LC‑BIO Technologies CO., LTD., Hangzhou, Zhejiang Province, China. DNA libraries were constructed using the TruSeq Nano DNA LT Library Prep Kit.

Raw sequencing reads were processed to generate valid reads for downstream analysis. First, sequencing adapters were removed using cutadapt v1.9. Second, low‑quality reads were trimmed with fqtrim v0.94 based on a sliding‐window algorithm. Third, reads were mapped against the host genome using bowtie2 v2.2.0 to filter host‐derived contaminating sequences. After quality filtering, clean reads from each sample were de novo assembled into metagenomic contigs using IDBA‐UD v1.1.1. Coding regions (CDS) on metagenomic contigs were predicted by MetaGeneMark v3.26. CDS sequences across all samples were clustered by CD‐HIT v4.6.1 to generate unigenes. Unigene abundance per sample was calculated as TPM values based on read counts mapped by bowtie2 v2.2.0. Taxonomic assignment for unigenes was performed via DIAMOND v0.9.14 alignment against the NCBI NR database for lowest common ancestor (LCA) annotation. Functional annotation of unigenes was obtained by searching against the eggNOG and KEGG databases. Based on taxonomic and functional annotation of unigenes as well as their abundance profiles, differential analysis was carried out at taxonomic, functional and gene‐wise levels using the Kruskal‐Wallis test, followed by Dunn's post‐hoc test for pairwise comparisons, for groups with biological replicates. Bioinformatic analyses were partly performed using OmicStudio tools (https://www.omicstudio.cn/tool). The sequencing data generated in this study have been deposited in the NCBI Gene Expression Omnibus (GEO) database under accession number GSE325164, and are publicly accessible at https://www.ncbi.nlm.nih.gov/geo/query/acc.cgi?acc=GSE325164.

### 4.10 Untargeted Metabolomics Analysis

The liquid chromatography tandem mass spectrometry (LC‐MS/MS) analysis of the metabolites of mouse feces was performed by the Thermo UHPLC‐Q Exactive HF‐X system in both positive‐ and negative‑ion electrospray ionization (ESI) modes at LC‐BIO Technology Co., Ltd. The acquired MS data were pre‐processed using XCMS software, including peak picking, peak grouping, retention time correction, second peak grouping, and annotation of isotopes and adducts. LC‑MS raw data files were converted into mzXML format and further processed by the XCMS, CAMERA and metaX toolboxes in R software. Each ion was identified by combining retention time (RT) and m/z information. Peak intensities were recorded, and a three‑dimensional matrix containing unique assigned peak indices (retention time‐m/z pairs), sample names (observations), and ion intensity values (variables) was generated. Metabolite annotation was performed by matching the exact molecular mass (m/z) against online KEGG and HMDB databases. A mass tolerance of 10 ppm was applied for database matching. Candidate metabolites were further validated for molecular formula according to isotopic distribution patterns. Raw metabolite intensities were normalized using the “medium” method. Hierarchical clustering was carried out with the pheatmap package. Supervised PLS‑DA was performed using the ropls R package, and variable importance in projection (VIP) values for each ion were computed. Differentially accumulated metabolites were screened by satisfying three criteria simultaneously: *P* < 0.05, fold‑change (FC) > 1.2, and VIP from PLS‑DA > 1.2. KEGG pathway enrichment analysis was conducted based on the hypergeometric test. Bioinformatic analysis was performed using the OmicStudio tools at https://www.omicstudio.cn/tool. The metabolomics data reported in this study are available in the NIH Common Fund's National Metabolomics Data Repository (NMDR; Metabolomics Workbench) with Study ID ST004751 and Project ID PR003017. The data can be accessed via the project DOI: https://doi.org/10.21228/M8PV94.

### 4.11 Fecal Microbiota Transplantation

Fecal microbiota transplantation (FMT) was performed as reported previously with slight modifications [16]. In brief, 8‐week‐old female donor mice (*n =* 5 per group) were orally administered with KU (100 mg kg^−1^) or vehicle for 2 weeks. Then, the stools from donor mice in each group were pooled, and 100 mg was resuspended in 1 mL of sterile saline. The solution was vigorously mixed and centrifuged at 800 g for 3 min. After inducing EAE in recipient mice and administering antibiotic cocktails (ABX) orally for one week, the supernatant was prepared from the feces of donor mice and administered orally to recipient EAE mice once a day (*n =* 9 per group). The ABX consisted of a mixture of antibiotics (antibiotics per 1 L of water: metronidazole 1 g, vancomycin 500 mg, neomycin 1 g, ampicillin 1 g).

### 4.12 ABX Treatment

Eight‐week‐old female C57BL/6 mice (*n =* 8 per group) were gavaged with 200 µL of ABX daily to deplete their gut microbiota for one week. Then, the mice were randomly assigned to four groups: EAE+vehicle group, EAE+ABX group, EAE+ABX+KU group, and EAE+ABX+KU+*A. muciniphila* group. KU or *A. muciniphila* (10^9^ CFU) administration was started from 11 to 21 dpi.

### 4.13 Network Pharmacology

The three‐dimensional structure of IAAld was obtained in SDF format from the PubChem database. Structural conversion to MOL2 format was performed using OpenBabel, and the resulting file was subsequently submitted to the Pharmapper server for potential target prediction. MS‐related targets were collected from the DrugBank, Therapeutic Target Database, and GeneCards databases, standardized against UniProt, and intersected with IAAld targets, yielding 94 intersection targets. Subsequently, KEGG pathway enrichment analysis was conducted. The *p‐*value and enrichment factor thresholds were set to screen for statistically significant pathways and to explore the potential molecular mechanism of IAAld intervention in MS.

### 4.14 Molecular Docking

Using the PubChem database (http://pubchem.ncbi.nlm.nih.gov/) to obtain the 2D structure of IAAld, the 2D structure was imported into ChemOffice to generate the 3D structure, and the resulting structure was saved as a mol2 file. The PDB format file of the AhR protein was obtained using the RCSB PDB database (http://www.rcsb.org/). The higher‐resolution crystal structure was selected as the molecular docking receptor. Pymol 2.6 was used to perform dewatering and phosphate removal on the protein, and the resulting structure was saved as a PDB file. The structures of proteins and small molecules were processed using Autodock 1.5.6. The proteins were hydrogenated, the ligands of small molecules were hydrogenated, the torsional forces were determined, and then the docking box coordinates were determined. Molecular docking was performed using AutoDockVina software to explore protein‐ligand interactions. The optimal docking conformation was screened with Binding Free Energy as the core index to evaluate the binding activities of the two.

### 4.15 Immunofluorescent Staining

Th1 and Th17 cells differentiated in vitro were inoculated on polylysine‐coated slides. After fixation with 4% paraformaldehyde for 15 min, permeabilization with 0.1% Triton X‐100 for 10 min, and room‐temperature blocking with 5% BSA for 1 h, the anti‐mouse AhR primary antibody was added and incubated overnight at 4°C. After PBS washing, the cells were then incubated with Alexa Fluor 488‐labeled goat anti‐mouse IgG secondary antibody at room temperature in the dark for 1 h. Subsequently, the staining was carried out in the dark with DAPI staining solution for 10 min, the plates were sealed with anti‐fluorescence quenchers, and the AhR fluorescence signals and nuclear localization were observed under a laser confocal microscope (Olympus FV3000).

### 4.16 Statistical Analysis

Statistical analysis was performed using GraphPad Prism 9 software. Comparisons between two groups were performed using the paired and unpaired Student's t‐test or Mann‐Whitney test. Differences in α‐diversity of microbiota between different groups were tested by one‐way analysis of variance (ANOVA) or the non‐parametric Kruskal‐Wallis test. The measurement data between different groups were compared using the one‐way or two‐way ANOVA with Bonferroni's post hoc test. Data are expressed as the mean ± SEM. *P <* 0.05 was considered statistically significant.

## Author Contributions

Conceptualization: **J.G**., **J.J.L**., and **K.X.R**.; Methodology: **X.Y.H**., **J.Q.D**., **Z.H.P**., **X.K**., **C.Z**., and **H.Z.L**.; Investigation: **X.Y.H**., **Z.H.P**., **W.T.H**., and **D.W**.; Visualization: **X.K**. and **Y.L**.; Supervision: **J.G**., **J.J.L**., and **K.X.R**.; Writing – original draft: **X.Y.H**., **J.Q.D**., and **Z.H.P**.; Writing – review & editing: **X.Y.H**., **J.Q.D**., **Z.H.P**., and **J.G**.

## Ethics Statement

All animal experimental procedures were approved by the Institutional Animal Care and Use Committee of The Fourth Military Medical University.

## Conflicts of Interest

The authors declare no conflicts of interest.

## Supporting information




**Supporting File**: advs78358‐sup‐0001‐SuppMat.docx.

## Data Availability

The data that support the findings of this study are available from the corresponding author upon reasonable request.
