# Supplementary material for: Gut Microbiota‐Derived Indole‐3‐Acetaldehyde Contributes to the Disease‐Alleviating Effects of Kurarinone in Experimental Autoimmune Encephalomyelitis
Source: Adv Sci (Weinh). 2026 Oct 15:e78358. Online ahead of print. doi: 10.1002/advs.78358 (PMC13673060; doi:10.1002/advs.78358)
Supplement: Supplementary file 1 [file ADVS-9999-e78358-s001.docx]

Supporting Information

**Gut microbiota-derived indole-3-acetaldehyde contributes to the disease-alleviating effects of kurarinone in experimental autoimmune encephalomyelitis**

*Xinyan Han^1,3^, Jiaqi Ding^1,3^, Zonghao Pan^1,3^, Weitai He^1^, Xue Kang^1^, Cong Zhao^2^, Yi Li^1^, Di Wu^1^, Hongzeng Li^1^, Kaixi Ren^1,^*, Jiaji Lin^1,^*, Jun Guo^1,4,^**

*Corresponding author. Email: [guojun_81@163.com;](mailto:guojun_81@163.com;) [timklin@foxmail.com;](mailto:timklin@foxmail.com;) [encore163@163.com](mailto:encore163@163.com)

**This PDF file includes:**

Supplementary Methods

Supplementary Figures 1 to 9

Supplementary Methods

DNA qPCR quantification

Genomic DNA was extracted from samples using Magnetic Bead‑based Soil & Stool Genomic DNA Extraction Kit (DP712, Tiangen Biotech) according to the manufacturer’s protocol. Purified DNA was 75‑fold diluted as qPCR template. Amplification was performed with SYBR qPCR Mix (DLQ101, Daling Biotechnology) in a 10 μL reaction system. The primer pairs were: ***A. muciniphila***: AM1‑F (5′‑CAGCACGTGAAGGTGGGGAC‑3′); AM2‑R (5′‑CCTTGCGGTTGGCTTCAGAT‑3′); internal reference (total bacteria): P338‑F (5′‑ACTCCTACGGGAGGCAGCAG‑3′); P518‑R (5′‑ATTACCGCGGCTGCTGG‑3′). Thermal profile: initial denaturation at 95 °C for 5 min; 40 cycles of 95 °C for 15 s, 60 °C for 20 s, 72 °C for 20 s (fluorescence collected at 72 °C), followed by melting-curve analysis. Relative A. muciniphila DNA abundance was calculated via the 2^−ΔΔCt^ method. Total bacterial DNA was used for normalization, and the EAE group was set as the calibrator.

LC-MS/MS Analysis

Brain tissue: Briefly, 200 mg wet mouse brain tissue was weighed and homogenized with grinding beads in 400 μL pre‑cold methanol. Samples were homogenized twice on ice, followed by 20 min incubation on ice for sufficient extraction. After centrifugation at 13000 g for 10 min at 4 °C, the total supernatant was collected and gently dried under nitrogen gas. The residue was reconstituted in 25 μL methanol, centrifuged at 13000 g for 10 min at 4 °C, and the supernatant was subjected to LC‑MS/MS analysis.

Fecal samples: 50 mg mouse feces were mixed with 500 μL pre‑cold methanol and ground by syringe. After centrifugation (13000 g, 10 min, 4 °C), the supernatant was collected. The pellet was re‑extracted with additional 250 μL pre‑cold methanol. The two supernatants were combined, dried under gentle nitrogen flow, reconstituted in 100 μL methanol, centrifuged, and filtered through a 0.22 μm PTFE membrane before LC‑MS/MS injection.

Serum samples: 200 μL mouse serum was mixed with 400 μL pre‑cold methanol for protein precipitation, incubated at -20 °C for 20 min, and centrifuged at 13000 g for 10 min at 4 °C. The resulting supernatant was dried by nitrogen, reconstituted in 25 μL methanol, vortexed and centrifuged for LC‑MS/MS detection.

*A. muciniphila* culture was centrifuged at 4000 g for 10 min at 4 °C, and the supernatant was filtered through a 0.22 μm membrane. 1 mL filtrate was dried under nitrogen flow in cold and dark conditions, then reconstituted with 100 μL ice-cold methanol. After centrifugation (12000 g, 4 °C), the reconstituted sample was analyzed by LC-MS/MS.

Calibration curve preparation: 1 mg IAAld standard was dissolved in 1 mL mass‑spectrometry‑grade methanol to prepare the stock solution. Serial dilutions were performed in methanol to obtain calibration working solutions at concentrations of 20 ng/mL, 100 ng/mL, 300 ng/mL, 1000 ng/mL and 2000 ng/mL.

Chromatographic separation was performed on an LC‑30AD liquid chromatography instrument equipped with an XSelect HSS T3 (5.0 μm, 4.6 × 100 mm, Waters, USA) column. The solvent system consisted of 0.1% formic acid in water (solvent A) and 0.1% formic acid in acetonitrile (solvent B). The gradient elution program was set as follows: 0-1.0 min, 10% B; 1.0-5.0 min, 10%-60% B; 5.0-8.0 min, 60%-95% B; 8.0-9.5 min, 95% B; 9.5-9.6 min, 95%-10% B; 9.6-12.0 min, 10% B. The column temperature was maintained at 40 °C, the flow rate was 0.4 mL/min, and the injection volume was 5 μL. Analytes were monitored by positive‑ion scheduled multiple‑reaction monitoring (Scheduled MRM) mode on an AB SCIEX API 4000 mass spectrometer coupled to LC‑30AD (Shimadzu, Japan). The operating parameters were as follows: collision gas (CAD), 8 psi; curtain gas (CUR), 20 psi; ion source gas 1 (GS1) and ion source gas 2 (GS2), 50 and 50 psi, respectively; ion spray voltage (IS), 4500 V; turbo spray temperature (TEM), 500 °C. Dwell time was set as 50-100 ms per channel. The MRM transition for IAAld was m/z 160.1-130.1 (loss of CH₂O, 30 Da), with declustering potential (DP) of 60 V, collision energy (CE) of 15 V, and collision cell exit potential (CXP) of 10 V.

Supplementary Figures

Figure S1. Gating strategy for flow cytometric analysis. Representative flow cytometric gating plots showing the sequential gating strategy: firstly, lymphocytes were gated based on FSC-A and SSC-A. Single cells were then selected by FSC-A and FSC-H to exclude doublets. CD4⁺ T cells were identified by FITC-conjugated anti-CD4 antibody staining, followed by gating for IL-17A⁺ (Th17) cells via APC-conjugated anti-IL-17A antibody and IFNγ⁺ (Th1) cells via PE-conjugated anti-IFNγ antibody.

Figure S2. Flow cytometric analysis of CD3⁺CD8⁺ T cells, CD3^-^CD19⁺ B cells, and CD3^-^NK1.1⁺ cells in the CNS and spleen. Representative flow cytometric plots and quantitative analysis of CD3^+^CD8⁺ T cells, CD3^-^CD19⁺ B cells, and CD3^-^NK1.1⁺ cells in the CNS (**A**) and SP (**B**) of the Ctrl, EAE, and KU groups. Data are presented as mean ± SEM. **P* < 0.05, ***P* < 0.01, ****P* < 0.001.

Figure S3. Analysis of gut microbial community composition. (**A**) Venn diagram showing the number of shared and unique operational taxonomic units (OTUs) among the Ctrl, EAE, and KU groups. (**B-F**) Stacked bar plot of gut microbial relative abundance at the phylum, class, order, genus, and species levels in the Ctrl, EAE, and KU groups.

Figure S4. KU promotes the proliferation of *A. muciniphila* *in vitro. A. muciniphila* was cultured in fluid thioglycollate (FT) medium supplemented with 1 μᴍ or 10 μᴍ KU. Bacterial CFU was determined at 0, 12, 24, 36, 48, 60 and 72 h. Data are presented as mean ± SEM. ****P* < 0.001 vs. Ctrl group.

**Figure S5. LC‑MS/MS detection of indole‑3‑acetaldehyde (IAAld).** (**A**) Representative MRM chromatogram of IAAld standard at 300 ng/mL, retention time = 4.06 min. (**B**) Standard calibration curve for quantitative determination of IAAld. The regression equation is y = 0.0039x - 40.526, with R² = 0.999.

**Figure S6. KU attenuates EAE and elevates IAAld levels in serum and brain.** (**A**) Clinical scores of vehicle‑treated EAE mice and KU‑treated EAE mice post‑immunization (*n* = 7 per group). (**B**) Disease incidence curves for EAE mice in the two groups. (**C**) Cumulative clinical scores and peak scores in each group. (**D**) qPCR‑measured relative DNA abundance of *A. muciniphila* in fecal samples. (**E**) LC‑MS/MS relative quantification of IAAld levels in serum (ng/mL) and brain tissues (ng/g). (**F**) Spearman correlation analysis among IAAld levels in serum, brain, relative DNA abundance of *A. muciniphila* and EAE clinical scores. Data are presented as mean ± SEM. **P* < 0.05, ***P* < 0.01, ****P* < 0.001.

**Figure S7. *A. muciniphila* supplementation elevates IAAld levels and ameliorates EAE in antibiotic‑treated mice.** (**A**) Clinical scores of EAE+ABX and EAE+ABX+*A. muciniphila* mice after immunization. Data are derived from the same experimental cohort presented in Figure 3. (**B**) LC‑MS/MS relative quantification of IAAld levels in feces (ng/g), serum (ng/mL) and brain tissues (ng/g). (**C**) Spearman correlation analysis among IAAld levels in feces, serum, brain and EAE clinical scores. Data are presented as mean ± SEM. **P* < 0.05, ****P* < 0.001.

**Figure S8. Exogenous IAAld administration increases IAAld concentrations in serum and brain.** LC‑MS/MS relative quantification of IAAld levels in serum (ng/mL) and brain tissue (ng/g) from EAE mice at day 21 post‑immunization, treated with vehicle or IAAld (80 mg/kg). Data are presented as mean ± SEM. ***P* < 0.01, ****P* < 0.001.

**Figure S9. KU alleviates EAE in an AhR-dependent manner.** (**A**) Clinical scores of EAE mice from EAE, EAE+KU (*i.g*., 100 mg/kg) and EAE+KU+AhRi (SR1, AhR inhibitor, *i.p*., 50 mg/kg) groups after immunization (*n* = 6 per group). (**B**) Disease incidence curves of EAE‑induced mice in the three experimental groups. (**C**) Quantification of cumulative clinical scores and peak score for each group. (**D**) Representative HE and LFB staining of spinal cord sections to evaluate inflammatory infiltration and demyelination, respectively. Scale bar, 200 μm; zoom‑in view, 100 μm. (**E**) Quantification of inflammation scores and demyelination scores of spinal cord tissues. (**F‑G**) Quantitative analysis of CD4⁺IFNγ⁺ (Th1) and CD4⁺IL‑17A⁺ (Th17) cell proportions in the CNS and spleen (SP). (**H‑I**) Representative flow cytometry plots showing CD4⁺IFNγ⁺ (Th1) and CD4⁺IL‑17A⁺ (Th17) cell populations isolated from the CNS and SP of different groups. Data are presented as mean ± SEM. **P* < 0.05, ***P* < 0.01, ****P* < 0.001.
